# The Effects of Ca^2+^ and MgADP on Force Development during and after Muscle Length Changes

**DOI:** 10.1371/journal.pone.0068866

**Published:** 2013-07-16

**Authors:** Fabio C. Minozzo, Dilson E. Rassier

**Affiliations:** 1 Department of Kinesiology and Physical Education, McGill University, Montreal, Quebec, Canada; 2 Department of Physiology, McGill University, Montreal, Quebec, Canada; 3 Meakins-Christie Laboratories, McGill University, Montreal, Quebec, Canada; 4 Department of Physics, McGill University, Montreal, Quebec, Canada; Semmelweis University, Hungary

## Abstract

The goal of this study was to compare the effects of Ca^2+^ and MgADP activation on force development in skeletal muscles during and after imposed length changes. Single fibres dissected from the rabbit psoas were (i) activated in pCa^2+^4.5 and pCa^2+^6.0, or (ii) activated in pCa^2+^4.5 before and after administration of 10 mM MgADP. Fibres were activated in sarcomere lengths (SL) of 2.65 µm and 2.95 µm, and subsequently stretched or shortened (5%SL at 1.0 SL.s^−1^) to reach a final SL of 2.80 µm. The kinetics of force during stretch were not altered by pCa^2+^ or MgADP, but the fast change in the slope of force development (P_1_) observed during shortening and the corresponding SL extension required to reach the change (L_1_) were higher in pCa^2+^6.0 (P_1_ = 0.22±0.02 P_o_; L_1_ = 5.26±0.24 nm.HS^.1^) than in pCa^2+^4.5 (P_1_ = 0.15±0.01 P_o_; L_1_ = 4.48±0.25 nm.HS^.1^). L_1_ was also increased by MgADP activation during shortening. Force enhancement after stretch was lower in pCa^2+^4.5 (14.9±5.4%) than in pCa^2+^6.0 (38.8±7.5%), while force depression after shortening was similar in both Ca^2+^ concentrations. The stiffness accompanied the force behavior after length changes in all situations. MgADP did not affect the force behavior after length changes, and stiffness did not accompany the changes in force development after stretch. Altogether, these results suggest that the mechanisms of force generation during and after stretch are different from those obtained during and after shortening.

## Introduction

Length changes imposed to activated muscles are commonly used to study the molecular mechanisms of contraction [1]–[6]. When muscle fibres are activated and subsequently stretched or shortened, force changes in four major steps: a fast rate of force change (phase 1) followed by a slower rate of force change (phase 2), after which the force stabilizes slowly (phase 3) to asymptotically return to a new steady state (phase 4) [4]–[6]. Although the mechanisms by which myosin crossbridges contribute to force changes observed during muscle stretch and shortening are still under investigation [7]–[12], phase 1 is commonly attributed to the elastic behavior of the crossbridges, and phase 2 is commonly associated with changes in the occupational fraction of crossbridges in the pre- and post- power-stroke states during the actomyosin cycle [5], [6].

After stretch or shortening, force stabilizes at a higher or a lower level than that produced during isometric contractions at corresponding lengths, respectively (phase 4); these phenomena have been referred to as residual force enhancement and force depression [13]–[15]. Attempts to correlate these last-longing changes in force with changes that happen *during* length changes have proved inconclusive. It has been suggested that stretch can induce changes in crossbridge kinetics leading to a long-lasting increase in the number of crossbridges attached to actin [16], and that shortening can cause crossbridge deactivation due to newly overlap zone formed between myosin and actin filaments [15], [17]. However, studies that measured stiffness – a putative measurement of crossbridges attached to actin – are contradictory; some show a direct relationship between stiffness and residual force changes [18], [19] and others fail to find any correlation between the two variables [14], [20].

The events dominating crossbridge kinetics during muscle stretch and shortening, and their potential relation with the residual force enhancement and depression need investigation. In this study, we approached this problem by investigating force during and after length changes (shortening and stretching) while altering either the population of attached crossbridges using different levels of Ca^2+^ activation, or biasing crossbridges into a strong-binding with actin by using MgADP activation [21]. Based on previous studies [6], [22], [23], we hypothesized that an increase in the number of crossbridges would not affect the relative forces produced during and after length changes, while changes in the crossbridges binding state would affect force during and after length changes. Confirmation of these hypotheses would link changes in crossbridge kinetics during length changes to the long-lasting effects observed in skeletal muscles.

## Methods

### Fibre Preparation

Muscle bundles (2–3 cm) from the rabbit psoas muscle were dissected, tied to wooden sticks, and chemically permeabilized as previously described [4], [5]. Muscles were incubated in rigor solution (pH = 7.0) for approximately 4 hours, then transferred to a rigor:glycerol (50∶50) solution for 15 hours, before storage in a fresh rigor:glycerol (50∶50) solution containing a cocktail of protease inhibitors (Roche Diagnostics, USA) in a freezer (-20°C) for at least seven days. Prior to the experiment, one muscle sample was transferred to a fresh rigor solution to be defrosted in the fridge (4°C) for one hour before use. After cutting a small section (∼4 mm in length) from the sample, single fibers were dissected in relaxing solution (pH = 7.0) and fixed with two aluminum foil clips. The fibres were placed inside a temperature-controlled chamber and attached between a force transducer (Model 400A, Aurora Scientific, Toronto, Canada) and a length controller (Model 312B, Aurora Scientific, Toronto, Canada). The protocol was approved by the McGill University Animal Care Committee (protocol # 5227) and complied with the guidelines of the Canadian Council on Animal Care.

### Solutions

The rigor solution (pH 7.0) was composed of (in mM): 50 Tris, 100 NaCl, 2 KCl, 2 MgCl_2_, and 10 EGTA. The relaxing solution used for muscle storage and dissection (pH 7.0) was composed of (in mM): 100 KCl, 2 EGTA, 20 Imidazole, 4 ATP and 7 MgCl_2_. The experimental solutions with pCa^2+^ of 4.5, 6.0 and 9.0 (pH 7.0) contained (in mM): 20 imidazole, 14.5 creatine phosphate, 7 EGTA, 4 MgATP, 1 free Mg^2+^, free Ca^2+^ in three concentration adjusted to obtain pCa^2+^ of 9.0, 6.0 and 4.5. KCl was used to adjust the ionic strength to 180 mM in all solutions. The final concentrations of each metal-ligand complex were calculated using a computer program based on Fabiato [24]. A pre-activating solution (pH 7.0; pCa^2+^9.0) with a reduced Ca^2+^ buffering capacity was used immediately prior to activation to minimize a delay in diffusion (in mM): 68 KCl, 0.5 EGTA, 20 Imidazole, 14.5 PCr, 4.83 ATP, 0.0013.7 CaCl_2,_ 5.41 MgCl_2_ and 6.5 HDTA. The MgADP solution was prepared by adding 0.214 g of MgADP to 50 mL of activation solution (pCa^2+^4.5), reaching a final MgADP concentration of 10 mM [15].

### Experimental Protocol

The average sarcomere length (SL) of the fibers in the experimental chamber was calculated in relaxing solution using a high-speed video system (HVSL, Aurora Scientific 901A, Toronto, Canada). Images from a selected region of the fibres were collected at 1000–1500 frames/second, and the SL was calculated using Fast Fourier Transform analysis based on the striation spacing produced by dark and light bands of myosin and actin, respectively. The fiber diameter and length were measured using a CCD camera (Go-3, QImaging, USA; pixel size: 3.2 µm×3.2 µm), and the cross-sectional area was estimated assuming circular symmetry.

Two separate sets of experiments were performed during this study: (i) fibres were activated in pCa^2+^4.5 and pCa^2+^6.0 (n = 13), or (ii) fibres were activated in pCa^2+^4.5 with or without MgADP (n = 7). Both sets of experiments followed the same procedures. Fibres were first activated to produce isometric contractions at nominal SLs of 2.65 µm, 2.80 µm and 2.95 µm. Fibres were subsequently activated at 2.65 µm and 2.95 µm, and a 5% SL change, at a speed of 1.0 SL.s^−1^ (stretch or shortening, random order) was imposed after full force development, obtaining a final SL of 2.80 µm in both cases. In all trials, control contractions at SL of 2.80 µm were elicited at the end of the experiments, and if isometric forces decreased by >15% in relation to the first isometric contraction, or when the striation pattern corresponding to the SL became unclear, the experiments were ended and the data was not used.

During all experiments, fibre stiffness (*K*) was assessed three times during the contractions: after force was fully developed, but before length changes (30 s after activation started), immediately after the change in length and before the force was stabilized (40.0075 s after activation started), and after the force was stabilized following the length changes (50 s after activation started). Stiffness was evaluated by applying a fast length step (dL = 0.3% L_o_) to the fibres, and dividing the change in force during this step by the length change (K = dF/dL) [25].

### Data Analysis

**Figure 1:**
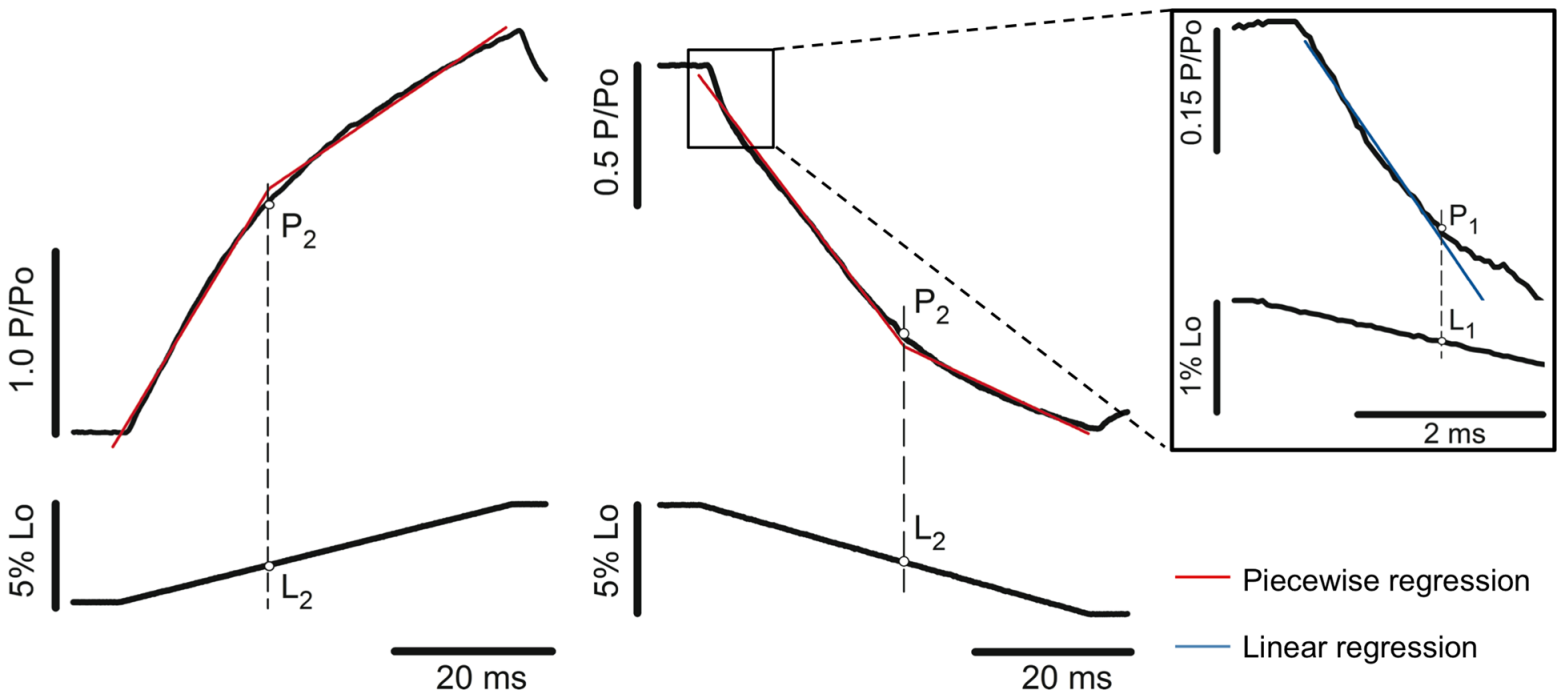
Detection of force transients during stretch and shortening of activated muscle fibres. Left-to-right: P_2_ and L_2_ detection during stretch and shortening, respectively. Force traces are on the top and fibre length variation at the bottom (P_o_ = isometric force; L_o_ = initial length). The force rise during stretch and the force decay during shortening can be fitted by two linear functions: *y*_1_ =  *a*_1_+ *b*_1_ × *xi* (restriction: *xi* ≤ *x*_o_) and *y*_2_ =  *a*_2_+ *b*_2_ × *xi* (restriction: *xi*>*x*_o_), where (*x*_o_, *y*_o_) represents coordinates of the critical transitions (coordinates for P_2_ in this case), *a*_1_ and *a*_2_ are the intercepts of the two regression lines, and *b*_1_ and *b*_2_ are the slopes of the two regression lines. The red traces in the graphs show the two-segmented piecewise regressions. The residual sum of squares (RSS) is based on the sum of the squares of each regression line: 
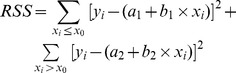
. RSS is used as a criterion to determine the optimal values of *a_1_, a_2_, b_1_, b_2_, and x_o_* - those belonging to the minimal RSS are considered optimal. The blue trace displayed on the inset correspond to a simple linear regression, based on the best fit for the force coordinates (*x*_o_, *y*_o_) during the first 2–3 ms, and P_1_ corresponds to the first data point where the regression does not follow the force trace. In both cases the statistic *F*-value and confidence intervals are calculated according to standard methods for regression analyses. L_1_ and L_2_ are extrapolated by crossing a perpendicular line passing by P_1_ and P_2_, respectively, until reaching the length traces.

The force and stiffness obtained after stretch, after shortening and during the isometric reference contractions were compared between different conditions from each set of experiment; (i) fibres activated in pCa^2+^4.5 and 6.0, and (ii) fibers activated in pCa^2+^4.5 with or without MgADP. The changes in the slope of force traces observed while fibres were stretched and shortened were detected using a two-segment piecewise regression, as previously described [4], [5]. The regression results were accepted when they presented a correlation coefficient (*r^2^*) >0.99. When this criterion was not met (a few cases for detection of P_1_), we fitted a single linear regression in the data points spanning from the first 2–3 ms in which force started to change, since force is linear during this time [5], [6]. The forces obtained at the first and second slope correspond to P_1_ and P_2_, and the SL amplitudes necessary to achieve P_1_ and P_2_ were named L_1_ and L_2_, respectively (Figure 1).

### Statistics

A two-way analysis of variance (ANOVA) for repeated measures was used to compare the forces and stiffness values in the different experiments (pCa^2+^ and MgADP conditions), before and after length changes. ANOVA for repeated measures was also used to compare the values of P_1_, P_2_, L_1_, and L_2_ obtained during shortening and stretch in the different sets of experiments. When significant changes were observed, post-hoc analyses for multiple comparisons were performed with Newman-Keuls tests. A level of significance of *P*≤0.05 was set for all analyses. All values are presented as mean ± S.E.M.

## Results

### Transient Forces during Length Changes

#### Experiments with different Ca^2+^ concentrations

**Figure 2:**
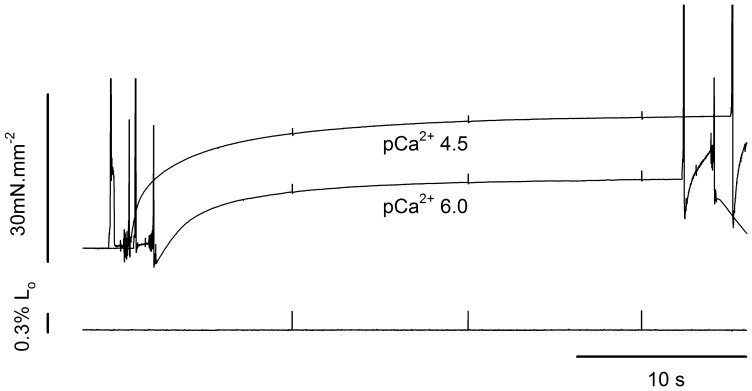
Superimposed isometric contractions in different Ca^2+^ concentrations. Sample records from typical isometric contractions produced in pCa^2+^4.5 and pCa^2+^6.0 (top), and corresponding length traces (bottom). The average sarcomere lengths in each contraction were 2.82 µm, and 2.83 µm, respectively.

Figure 2 shows two isometric contractions produced during a typical experiment in which a fibre was activated in pCa^2+^4.5 and subsequently in pCa^2+^6.0. The force produced in pCa^2+^4.5 is in the range observed in previous studies that used permeabilized fibers from mammalian muscles activated at low temperatures (4–5°C). These studies show forces ranging from 48 to 58 mN/mm^2^ when fibres contract at the plateau of the force-length relation [26]–[29]. Since we activated fibers on average sarcomere lengths between 2.7 µm and 2.9 µm, the force should be 20–30% lower than that produced at the plateau of the force-length relation, and thus our values are in close agreement with the literature. The force decreased when fibres were activated in pCa^2+^6.0 by 53.3±5.5%, similar to what has been reported in previous studies [4], [5].

**Figure 3:**
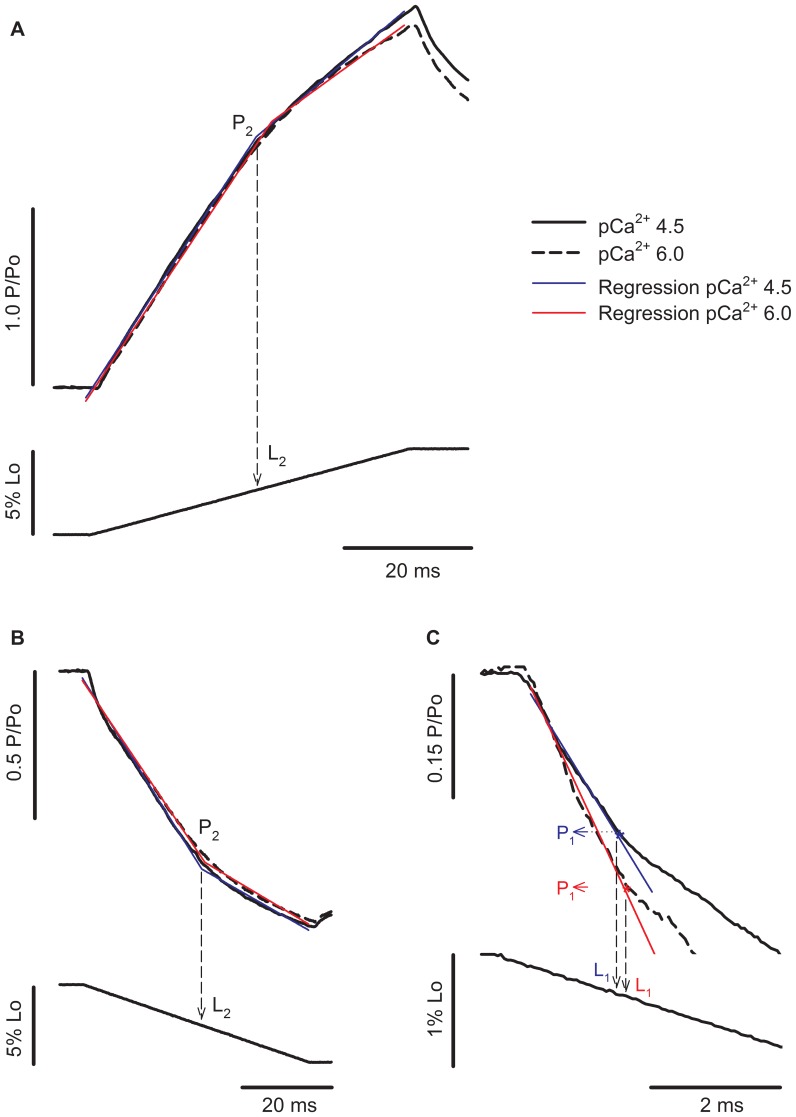
Force transients during length changes in different Ca^2+^ concentrations. (A) Superimposed contractions showing the force increase during stretch while the fibre was activated in pCa^2+^4.5 (solid line) and pCa^2+^6.0 (dashed line) (top), with corresponding changes in fibre length (bottom). All forces were normalized by the isometric forces (P_o_) before the stretch. The regression lines for the contractions produced in pCa^2+^4.5 and pCa^2+^6.0 are shown in blue and red, respectively. (B) Superimposed contractions showing the force decrease during shortening when a fibre was activated in pCa^2+^4.5 (solid line) and pCa^2+^6.0 (dashed line) (top), with the corresponding length changes (bottom). (C) Closer view from the initial shortening phase, showing that decreasing Ca^2+^ concentration induced an increase in L_1_ and P_1_ amplitudes. All forces were normalized by the isometric forces (P_o_) before the shortening. The regression lines for the contractions produced in pCa^2+^4.5 and pCa^2+^6.0 are shown in blue and red, respectively. Decreasing Ca^2+^ concentration increased L_1_ and P_1_ amplitude.

Figure 3 shows force and length traces recorded when a fibre was stretched or shortened in two different Ca^2+^ concentrations. The force was normalized by the maximum isometric force produced just before the stretch. The values of P_2_ of 2.42 P_o_ during stretch and 0.34 P_o_ during shortening observed in this fibre, when activated in pCa^2+^4.5, are in agreement with previous studies [3], [6], [12], [30]. The value of L_2_ observed during stretch was comparable with previous studies [31], [32], but higher than what we observed before, where sarcomeres needed to be stretched by ∼14 nm.HS^−1^ before a change in the force trace was observed [4], [33]. The difference may be related to the speed of stretch and SL, as in the previous study we used faster stretches at shorter SLs than used in the current experiments.

During stretch, P_2_ and L_2_ were not different between contractions performed in different pCa^2+^ (Figure 3A). We were unable to detect P_1_ during stretch. However, during shortening, the P_1_ amplitude (absolute distance between the P_1_ and P_o_, normalized by P_o_) and L_1_ were significantly higher in pCa^2+^6.0 than in pCa^2+^4.5 (Figure 3C). Note that the force does not reach a complete steady state just after shortening. The reasons for such behavior may vary, but it is likely associated with sarcomere length non-uniformities that develops with loaded shortening during full fiber activation and/or thin filament deactivation [6], [34].

**Figure 4:**
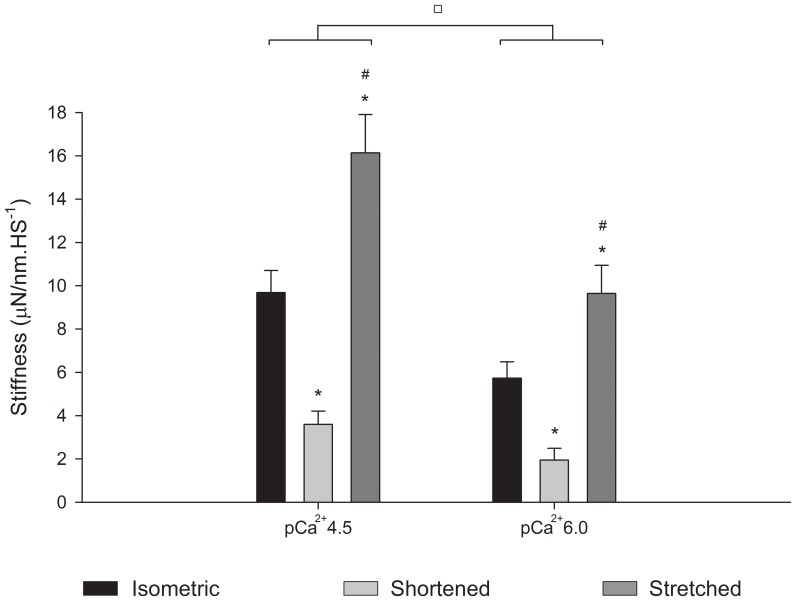
Mean stiffness values during length changes. Mean stiffness values (± S.E.M) during isometric contractions (black bar), shortening (light grey bar) and stretch (dark grey bar) from experiments performed in pCa^2+^4.5 and pCa^2+^6.0 (n = 13). Decreasing Ca^2+^ concentration significantly decreased the stiffness in all conditions. In both pCa^2+^, stiffness increased during stretch and decreased during shortening. * Significantly different from isometric,^ #^ significantly different from shortening, ^□^ groups significantly different from each other.

The stiffness decreased during shortening and increased during stretch (Figure 4). The relative stiffness increased less than the force during stretch in pCa^2+^4.5 and pCa^2+^6.0, but decreased similarly during shortening in both situations.

#### Experiments with MgADP

**Figure 5:**
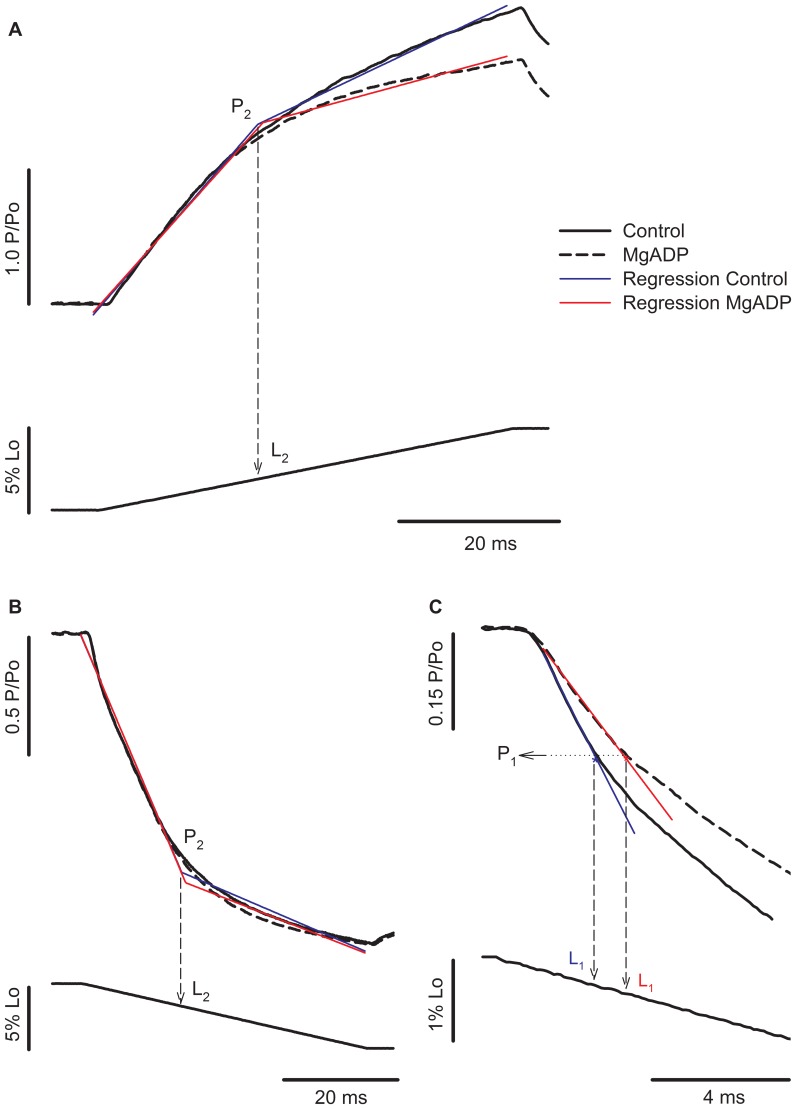
Force transients during length changes in fibres treated with MgADP. (A) Superimposed contractions showing the force increase during stretch (top) when the fibre was activated in pCa^2+^4.5 (solid line) and treated with MgADP (dashed line). The corresponding length changes are show in the bottom panels. All forces were normalized by the isometric forces (P_o_) before the stretch. The regression lines for the contractions produced in pCa^2+^4.5 and in presence of MgADP are shown in blue and red, respectively. (B) Superimposed contractions showing the force decrease during shortening (top) when a fibre was activated at pCa^2+^4.5 (solid line) and treated with MgADP (dashed line). The corresponding changes in fibre length are shown in the bottom. (C) Closer view from the initial shortening phase showing that MgADP activation induced an increase in L_1_. All forces were normalized by the isometric forces (P_o_) before the shortening. The regression lines for the contractions produced in pCa^2+^4.5 before and after MgADP treatment are shown in blue and red, respectively. MgADP treatment increased L_1_ significantly, while it did not change the other variables.

**Figure 6:**
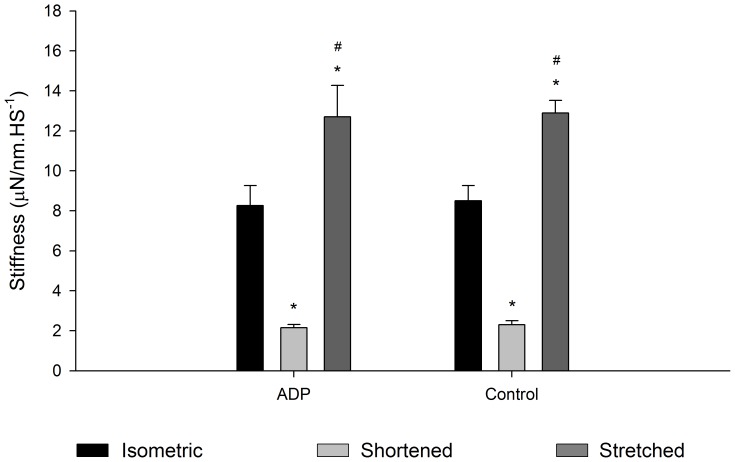
Mean stiffness values during length changes. Mean stiffness values (± S.E.M) during isometric contraction (black bar), shortening (light grey bar) and stretch (dark grey bar) obtained in experiments performed in pCa^2+^4.5 before and after MgADP treatment (n = 7). MgADP did not affect stiffness, but in stiffness was always increased during stretch and decreased during shortening. * Significantly different from isometric contractions,^ #^ significantly different from shortening.

Figure 5 shows force and length traces recorded when a fibre was stretched before and after MgADP treatment. The force was normalized by the maximum isometric force produced before the stretch. P_2_ and L_2_ were not different between the two contractions (P_1_ and L_1_ were undetectable during stretch) (Figure 5A). Figure 5B and 5C shows force and length traces recorded during fibre shortening. L_1_ was longer with the presence of MgADP, while all the other variables were not changed by MgADP (Figure 5C). The stiffness during length changes decreased during shortening and increased during stretch (Figure 6).

Table 1 summarizes the force and length transient values obtained in the two sets of experiments conducted in this study. Overall, during shortening P_1_ decreased and L_1_ increased at low Ca^2+^ concentration, whereas only L_1_ increased when fibres where treated with MgADP.

**Table 1:** Force transients and respective half-sarcomere length extensions during stretch and shortening.

Length changes	Experiments		P_1_	P_2_	L_1_	L_2_
**Stretch**	**i) Effects of Ca^2+^**	pCa^2+^4.5	–	2.51±0.19	–	29.83±1.45
		pCa^2+^6.0	–	2.60±0.14	–	30.37±1.81
	**ii) Effects of MgADP**	Control (pCa^2+^4.5)	–	2.48±0.14	–	27.33±0.70
		MgADP	–	2.47±0.21		25.28±1.71
**Shortening**	**i) Effects of Ca^2+^**	pCa^2+^4.5	0.85±0.02	0.28±0.04	4.48±0.25	29.51±1.67
		pCa^2+^6.0	0.78±0.02*	0.19±0.07	5.26±0.17*	30.17±1.49
	**ii) Effects of MgADP**	Control (pCa^2+^4.5)	0.80±0.03	0.09±0.14	4.34±0.19	26.77±0.94
		MgADP	0.78±0.04	0.17±0.09	5.52±0.20*	26.73±0.74

**Legend:** (i) Experiments performed with fibres activated in different Ca^2+^ concentrations (n = 13). (ii) Experiments performed with fibres treated with MgADP (n = 7). P_1_ and P_2_ correspond to the force/isometric force (P/P_o_). L_1_ and L_2_ correspond to the half sarcomere extension obtained at P_1_ and P_2_, and are given in nm.HS^−1^. * Significantly different from control.

### Residual Force Enhancement and Depression

#### Experiments with different Ca^2+^ concentrations

**Figure 7:**
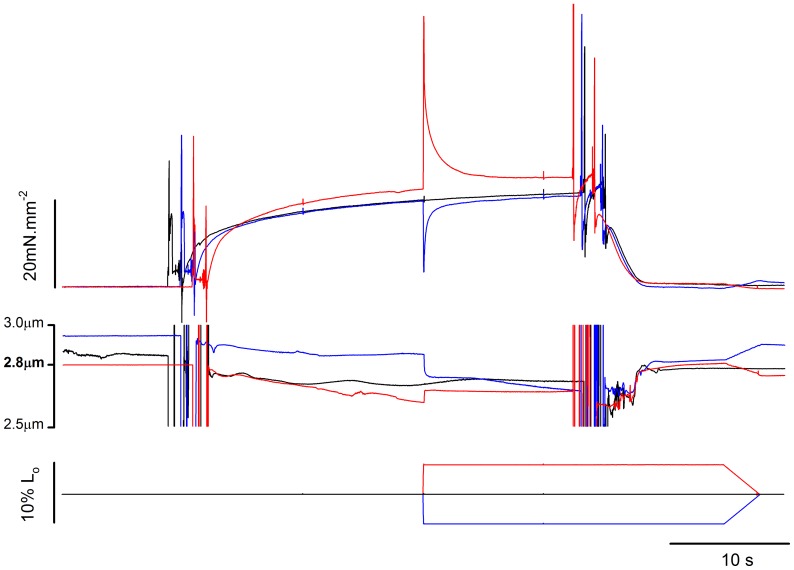
Typical experiment for analysis of residual force enhancement and force depression performed in pCa^2+^4.5. Superimposed force traces (upper panel), SL traces (mid panel), and length traces (lower panel) from a fibre activated in pCa^2+^4.5 and kept isometric (in black), stretched (in red), or shortened (in blue).

**Figure 8:**
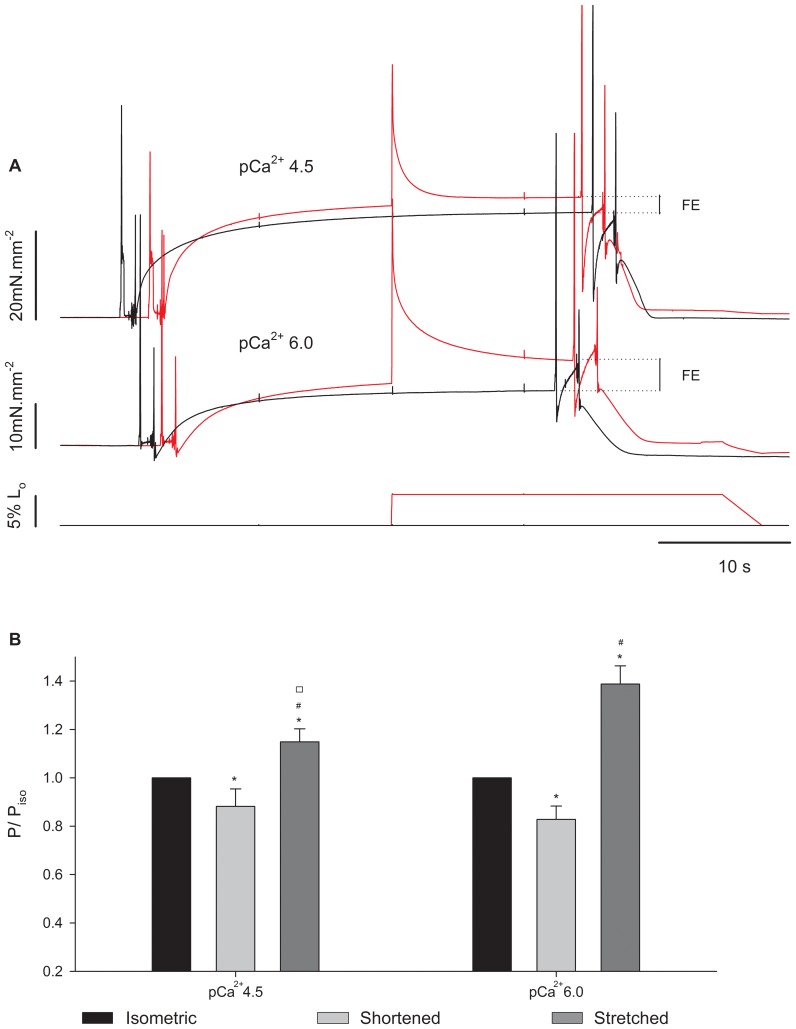
Residual force changes in different Ca^2+^ concentrations. (A) Sample records from a typical experiment showing superimposed contractions produced by a fibre activated in pCa^2+^4.5 and pCa^2+^6.0. Black: isometric contraction at SL of 2.8 µm. Red: isometric contraction at SL of 2.65 µm followed by a 5% stretch. The corresponding length changes are shown in the lower panels. (B) Mean (± S.E.M.) relative forces from fibres activated in pCa^2+^4.5 and pCa^2+^6.0 (n = 13) in three conditions: isometric (black bar), shortened (light grey bar) and stretched (dark grey bar). P = forces recorded 10 s after length changes; P_iso_ = force at the respective isometric condition. * significantly different from isometric,^ #^ significantly different from shortening, ^□^ significantly different from the same condition (shortened) in pCa^2+^6.0.

Figure 7 shows an experiment with three superimposed contractions produced in pCa^2+^4.5 in different SLs. The steady-state isometric forces after stretch and shortening were higher and lower, respectively, than the force produced during the isometric contraction at the corresponding length, as shown before [6],[14],[18],[35],[36]. Changing Ca^2+^ concentration changed the levels of force enhancement (Figure 8A), such that the increase in force was higher when the fiber was activated and stretched in pCa^2+^6.0 than in pCa^2+^4.5. This result was confirmed statistically; although the absolute force enhancement (4.2±0.8 mN.mm^−2^) was independent from pCa^2+^, the relative enhancement was higher in pCa^2+^6.0 (38.8±7.4%) when compared to pCa^2+^4.5 (14.9±5.6%). Changing Ca^2+^ concentration did not change the effects of shortening on the long-lasting force; the residual force depression was similar in pCa^2+^4.5 and pCa^2+^6.0 (Figure 8B).

**Figure 9:**
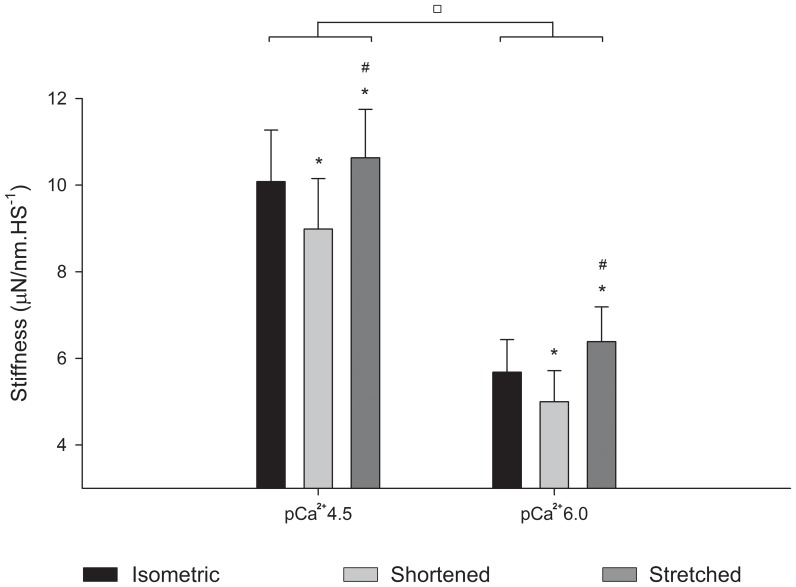
Mean stiffness after length changes. Mean stiffness values (± S.E.M) measured during isometric contractions (black bar), and when forces reach a new steady state after shortening (light grey bar) and stretch (dark grey bar) in experiments performed in pCa^2+^4.5 and pCa^2+^6.0. Decreasing Ca^2+^ concentration decreased the stiffness in all conditions. In both Ca^2+^ concentrations, the stiffness during stretch and shortening was higher and lower, respectively, than during isometric contractions. * Significantly different from isometric,^ #^ significantly different from shortening,^ □^ significantly different from each other.

In order to test if the changes in the residual force as a result of pCa^2+^ could be attributed to variations in the number of crossbridges attached to actin, we compared the stiffness after length changes and during isometric contractions. Although the absolute stiffness was higher in pCa^2+^4.5 (Figure 9), the stiffness enhancement after stretch was significantly larger in pCa^2+^6.0 (15.7±0.08%) than in pCa^2+^4.5 (6.4±0.03%). The level of stiffness depression after shortening was similar (12.3±2.70%) in both Ca^2+^ concentrations.

#### Experiments with MgADP

**Figure 10:**
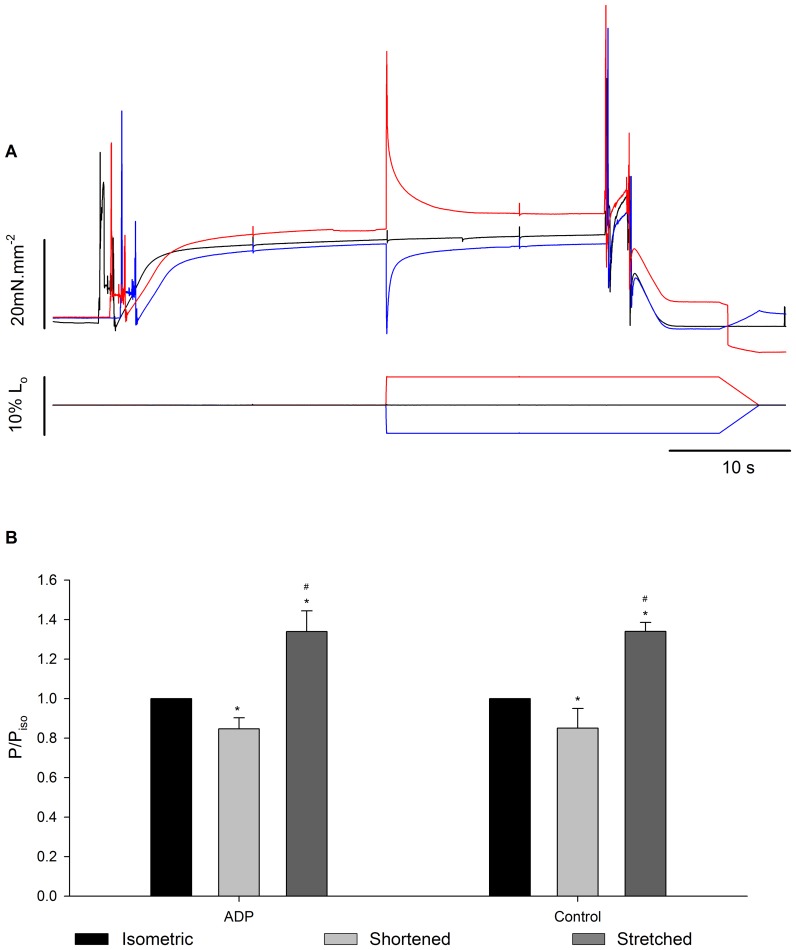
Residual force changes after MgADP treatment. (A) Sample records recorded during a typical experiment in a fibre activated in pCa^2+^4.5 with 10 mM MgADP. Black: isometric contraction at SL of 2.8 µm. Red: isometric contraction at SL of 2.65 µm followed by a 5% length stretch. Blue: isometric contraction at SL 2.95 µm followed by a 5% length shortening. Lower panel: correspondent fibre length changes. (B) Mean (± S.E.M.) forces produced by fibres (n = 7) activated in pCa^2+^4.5 and pCa^2+^4.5+10 mM MgADP during isometric contractions (black bar), after shortening (light grey bar) and after stretch (dark grey bar). P = forces 10 s after length changes. P_iso_ = force produced during the respective isometric condition. * significantly different from isometric,^ #^ significantly different from shortening.

**Figure 11:**
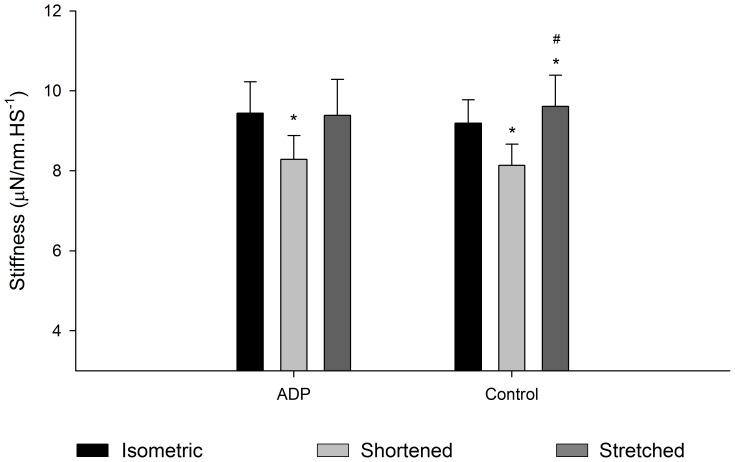
Mean stiffness after length changes. Mean stiffness values (± S.E.M) measured during isometric contractions (black bar), and when forces reach a new steady state after shortening (light grey bar) or stretch (dark grey bar) during experiments performed in pCa^2+^4.5 with and without MgADP (n = 7). * significantly different from isometric,^ #^ significantly different from shortening.

**Figure 12:**
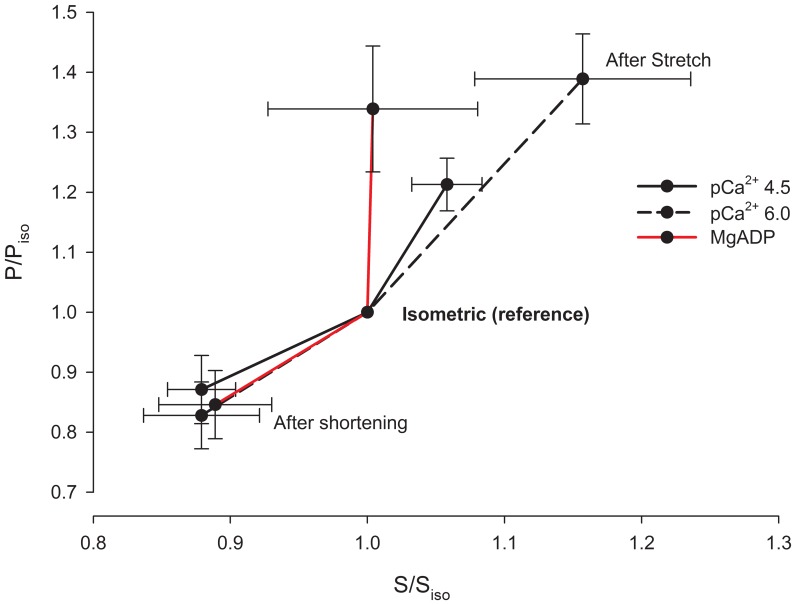
Relation between force and stiffness after length changes. Mean forces (P/P_iso_ +S.E.M.) plotted against the mean stiffness (S/S_iso_ ± S.E.M.). Relative stiffness in MgADP after stretch does not follow the same trend as in Ca^2+^ solutions. P = force measured 10 s after length changes. P_iso_ = force at the respective isometric condition; S = stiffness measured 10 s after length changes in every condition, S_iso_ = stiffness at the respective isometric condition.

Figure 10A shows a typical experiment with three superimposed contractions produced in different SL by a fibre activated in pCa^2+^4.5, in a solution containing 10 mM MgADP. As in the case of the experiments using different pCa^2+^, the forces produced after stretch and shortening were higher and lower, respectively, than the force produced during isometric contraction. MgADP did not affect the residual force enhancement or the force depression (Figure 10B). However stiffness after stretch did not increase in the presence of MgADP (Figure 11). When all experiments (Ca^2+^ and MgADP) are pooled, the relation between the history-dependence of force and stiffness is apparent (Figure 12), and shows that stiffness is affected by MgADP in the force enhanced state.

The stiffness measurements may be affected by compliance of the sarcomeres, and most specifically the compliance of the filaments. Although filament compliance may account for ∼50% of the sarcomere compliance [37], its actual contribution to the strain-force relationship in a half-sarcomere is unknown. Isolated thick and thin filaments stretched from zero force to maximal physiological force develop strains of 0.3% and 1.5%, respectively [38], [39], values that are too low for account for the changes in stiffness that we observed in this study. Most importantly, even if the compliance affects slightly the stiffness values that we obtained, there is no evidence that changes in Ca^2+^ concentration and MgADP affects the compliance of the filaments, and therefore the comparisons made throughout our study remain valid.

#### P_2_-to-peak/valley amplitude

**Figure 13:**
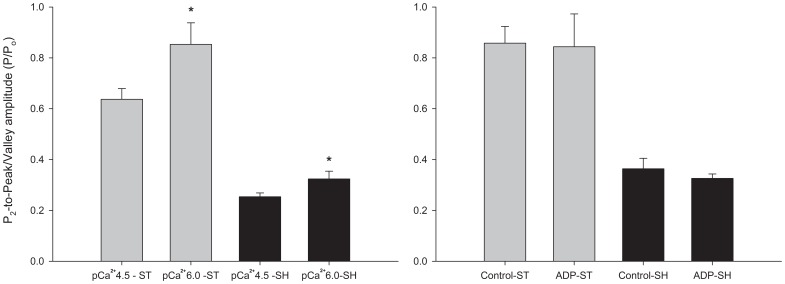
Difference between P_2_ and forces at the end of length changes (peak for stretching and valley for shortening). Left panel: experiments performed in different pCa^2+^. Right panel: experiments performed with MgADP. ST = P/P_o_ during stretch (light gray bar), SH = P/P_o_ during shortening (black bar). * Significant difference between two groups.

In order to analyze the relationship between the force increase and decrease after stretch or shortening during phase 3 and the residual forces after length changes, we measured the relative distances between P_2_ and the peak force during stretch, and between P_2_ and the lowest force (valley) obtained during shortening. We noticed these distances were significantly greater in pCa^2+^6.0 than in pCa^2+^4.5, but they were not affected when MgADP was added to the solution (Figure 13).

## Discussion

The main results of this study were that decreasing Ca^2+^ concentrations decreased the force produced during shortening and increased the force produced after stretch; these changes were accompanied by similar changes in stiffness. MgADP affected stiffness during shortening and after stretch, without concomitant changes in force. These results suggest that the mechanisms of force changes during stretch and shortening are different from those observed after the length changes, when forces have stabilized in a new steady-state.

### Transient Forces during Length Changes

We detected differences in P_1_ and L_1_ resulting from changing Ca^2+^ concentrations during shortening, as previously shown (Minozzo et al, 2012). P_1_ changes have been attributed mainly to crossbridges engaging in specific states of the power-stroke during shortening [5], [6], [12]. In a previous study [5], we developed a model to better understand the effect of crossbridges biased into myosin.ADP.Pi (pre-power-stroke) complex on P_1_. We observed that biasing crossbridges towards a pre-power-stroke state caused a decrease in isometric force and a decrease in P_1_ during shortening. Although the experimental data in that study also showed a trend to decrease P_1_ (figure 10 from Minozzo et al, 2012), the result was not statistically significant. In the current study, we observed that P_1_ amplitude and L_1_ were increased in pCa^2+^6.0. At lower Ca^2+^ concentrations, the absolute population of non-attached crossbridges is expected to be high; this population can affect the transition between non-force generating to force generating crossbridges via a cooperativity mechanism, which is more prominent in low than in saturating Ca^2+^ concentrations [40]. This interpretation is consistent with the experiments that showed a direct relationship between stiffness and P_1_ at different shortening velocities [6].

MgADP altered the relation between force development and stiffness during shortening by increasing only L_1_, decreasing the stiffness/force ratio. MgADP not only induces myosin strong-binding to actin [41], which in turn would “turn-on” adjacent actin binding sites leading to myosin cooperativity [42], but also decreases the rigor stiffness without concomitant changes in force [43].

P_2_ and L_2_ during shortening were not affected by lowering the Ca^2+^ concentration. Assuming that P_2_ and L_2_ represent the critical force and length, respectively, at which crossbridges detach during shortening, our results are consistent with the rationale that the number of crossbridges formed before shortening does not change the relative strain necessary for their detachment [5].

The values of P_2_ and L_2_ were not affected by Ca^2+^ concentration during stretch. While one previous study showed an increase in P_2_ when Ca^2+^ was elevated [4], and associated the increase to an enhanced stiffness/force ratio, another study [44] showed an increase in the stretch forces when Ca^2+^ was lowered from pCa^2+^4.5 to pCa^2+^6.3; these authors linked their result to a greater reliance on the rate of crossbridge detachment in low Ca^2+^ concentrations. Since the relative stiffness changes were not affected by Ca^2+^ concentrations, the stiffness increase during stretch did not follow the force increase, suggesting the later does not occur due to an increase in the number of crossbridges attached to actin. Instead, it suggests that the force increase during stretch can be caused either by an increase in force produced per crossbridge, or by pre-power-stroke crossbridges resisting to stretch [4], [11]. Therefore our results are in line with our working hypothesis, which mainly attributes force changes during stretch to crossbridges in a pre-power-stroke state. These crossbridges would not contribute to isometric force generation, but would have the capacity to resist to stretch.

### Residual Force Enhancement

Contrary to our hypothesis, the residual force enhancement was ∼2.6-fold larger in pCa^2+^6.0 than in pCa^2+^4.5. The effect of activation on the residual force enhancement is unclear. Rassier et al. [16] did not observed changes in the force enhancement in intact fibres isolated form the frog and activated with different frequencies of stimulation. However, Campbell and Moss [45] observed a greater relative force increase in a second stretch when two consecutive stretches were applied in pCa^2+^6.2 compared to pCa^2+^4.5. The authors suggested that the rate at which cycling crossbridges reach a steady state is considerably longer at lower Ca^2+^ concentrations. Considering that the residual force enhancement could be caused partially by an increase in the number of attached crossbridges, fibres activated at lower Ca^2+^ concentration would have a greater population of crossbridges available to attach after stretch, which in turn could be responsible for a higher force enhancement in lower Ca^2+^ concentrations.

If it is assumed that an increase in stiffness may be caused by an increase in the number of crossbridges attached to actin, MgADP might have eliminated this effect. Thus, a complementary mechanism to explain the increased residual force enhancement in low Ca^2+^ concentration must exist, since the stiffness did not increase as much as the force after stretch. Force enhancement has been also associated with activation of passive structures during stretch [46]–[50]. Titin stiffness may increase with increasing Ca^2+^ concentrations [51], leading to an increased force produced during stretch. While some studies showed an increased force when fibres lacking myosin-actin interaction were activated with Ca^2+^ [47], [51] other studies [52], [53] showed that titin may decrease stiffness with increasing Ca^2+^ concentration. Nevertheless, it is still temping to speculate that the increase in titin stiffness with Ca^2+^ contributed to force enhancement.

Finally, we cannot exclude that the residual forces after length changes may happen due to sarcomere/half-sarcomere length non-uniformity [50], [54], [55], a mechanism that would be also in line with the differences found when fibres were activated in different pCa^2+^. Accordingly, activation would lead to a population of sarcomeres that would elongate more, decreasing their amount of overlap until passive forces would emerge, while other sarcomeres would elongate less. The “weak” sarcomeres that would continuously elongate would reach a tension borne by passive elements that would equal the tension of the “strong”, shorter sarcomeres. It has been shown that myofibrils stretched at low Ca^2+^ concentrations presented more sarcomeres “yielding” (weakening) than at high Ca^2+^ concentration [56]. Assuming the same happened in our experiments, activation in pCa^2+^6.0 could have generated more sarcomere non-uniformity, causing a greater force enhancement than fibres activated in pCa^2+^4.5.

### Residual Force Depression

The changes in stiffness accompanied the changes in force after shortening, suggesting a decrease in the number of attached crossbridges, as previously observed [18]. Force depression has been attributed to crossbridge inhibition caused by angular distortion of the actin binding sites induced by shortening after activation [15], [17]. Changing Ca^2+^ concentrations did not change the residual force depression. This confirms our hypothesis that the number of crossbridges attached previously to shortening would not change the relative levels of force depression since the crossbridge inhibition mechanism is mainly dependent on the amount of new overlap zone formed between the two filaments after shortening.

The mechanisms proposing an inhibition of crossbridges attached in a newly overlap zone was strengthened in a previous study that we performed with isolated myofibrils (Pun et al. 2010). In that study the levels of force depression were decreased by MgADP activation, suggesting that activation via cooperativety counteracted part of the crossbridges inhibition caused by shortening. Differently, in the present study the residual force depression was not affected by MgADP activation. In the current study fibres were activated in saturating Ca^2+^ levels together with higher levels of MgADP (∼4 times higher than in the previous study). In this situation, the role of myosin cooperativity in the force inhibition is likely decreased, as MgADP competes with MgATP for the myosin binding site; the more ATP the lesser the influence of MgADP and cooperativity on fibre activation [41]. The reason MgADP was added to Ca^2+^ solution in the present study was that we did not want only to work with activation via cooperativity - in fact, we were interested in biasing a relatively large proportion of crossbridges into strong-bound states, expecting an opposite effect to what was previously observed with blebbistatin [4], [5].

### Relation between the Transient Force and the Residual Forces

The relation between the force during and after length changes (phase 4) remains unclear. Edman and Tsuchiya [57] found a strong relationship between the level of force increase after P_2_ and the level of residual force enhancement. They interpreted this first increase to a damping effect of “weak sarcomeres” working in parallel to elastic elements - this increase would be attenuated due to the release of elastic strain, but not cease after stretch. Assuming that fibres activated in low Ca^2+^ would allow more sarcomere “weakening" during and after stretch than at high Ca^2+^ concentration, one would expect that force increase after P_2_ would also be increased when Ca^2+^ concentration is lowered. Conversely, assuming that MgADP did not affect SL non-uniformity, it would not alter the relation between these two phases of force increase. We found that the levels of force enhancement after P_2_ were accompanied by similar levels of force enhancement at different Ca^2+^ concentrations and when fibres were treated with MgADP. Nevertheless, the relative force decline during shortening was more pronounced at pCa^2+^6.0 than in pCa^2+^4.5. Considering thin filament deactivation the main mechanism for residual force depression [17], force decline after P_2_ could only be partially attributed to this mechanism (see Roots et al. 2007). Apparently, distinct mechanisms are responsible also for force behavior after P_2_ in stretch and shortening.
